# Lead Poisoning, from the Industrial, Medical, and Social Points of View

**Published:** 1917-07

**Authors:** 


**Lead Poisoning, from the Industrial, Medical, and Social Points of View.** By Sir Thomas Oliver, M.A., M.D., F.R.C.P. Pp. viii. 294. London: H. K. Lewis, 1914. Price 5s. net.

These lectures, delivered at the Royal Institute of Public Health, are a singularly interesting account by the first authority on the subject of the way in which lead gains entrance to the human body in various trade processes, the effects of the metal on the organism, and how these effects may be got rid of. We are shown too how the dangers have been lessened by legislative measures and inspection, and the actual Factory and Workshop Orders in force are given in an appendix. Dust appears to be the most common source of plumbism, but even in this form the lead gets chiefly into the alimentary canal rather than into the lungs. The author does not regard the presence of basophilia in the stained blood as a reliable test, in spite of the great claims made for it, and he discusses with much care the evidence for the Wasserman reaction, which has been found in some cases of plumbism, and especially where pseudo-general paralysis exists, bnt the question does not appear to be finally settled.

Another interesting discussion too is that as to the effect of lead on the blood pressure. It appears to be certain that in those who have long worked in lead, there is a general tendency towards a rise above the normal and consequent risk of cerebral hemorrhage, but this does not occur as a rule in the earlier years of their work, while in acute poisoning there is often a great fall from the action of lead on the heart.

A very difficult question arises as to what constitutes lead poisoning from the legal point of view. Thus lead may be present in the body without causing any symptoms, just as a typhoid carrier may be in perfect health. The presence of lead in the urine is a reliable proof that it exists in the organism and is being eliminated, but the patient may show no ill-effects until some check to elimination suddenly produces serious symptoms. Are such persons suffering from lead poisoning before the paralysis occurs? Again, how can we decide whether gout or contracted kidney is or is not due to employment in lead work ?

In the chapter on treatment we find actual proof of the removal of lead from the organism by the double electric (galvanic current) bath. This is shown both by the measurement of the quantity of lead actually removed, and by the practical effect in stopping paralysis and colic.

